# Causes and types of voluntarily reported unintentional medication discrepancies in care transitions: a cross-sectional study

**DOI:** 10.1007/s11096-026-02189-x

**Published:** 2026-07-18

**Authors:** Betsie Limmen, Joy van Broekhuizen, Rob Essink, Judith de Ruijter-van Dalem, Linda van Eikenhorst, Patricia M. L. A. van den Bemt, Fatma Karapinar-Çarkit

**Affiliations:** 1 Department of Clinical Pharmacy and Toxicology, Maastricht University Medical Center+, PO Box 5800, 6202AZ Maastricht, The Netherlands; 2 Department of Clinical Pharmacy, CARIM Cardiovascular Research Institute Maastricht, Maastricht University, Maastricht, The Netherlands; 3 Department of Clinical Pharmacy and Pharmacology, University Medical Centre Groningen, Groningen, The Netherlands; 4 Dutch Institute for Rational Use of Medicine, Utrecht, The Netherlands; 5 Department of Clinical Pharmacy, NUTRIM Institute of Nutrition and Translational Research in Metabolism, Maastricht University, Maastricht, The Netherlands; 6 Department of Organization and Quality of Care, Netherlands Institute for Health Services Research (NIVEL), Utrecht, the Netherlands; 7 Department of Clinical Pharmacy, University Medical Centre Utrecht, Utrecht, The Netherlands

**Keywords:** Human error, Risk Management, Medication Reconciliation, Medication safety, Transitional care

## Abstract

**Introduction:**

Medication incident reporting systems enable healthcare professionals to report incidents and their analysis may help prevent reoccurrences. While previous research has primarily focused on medication errors in general, incidents involving unintentional medication discrepancies remain unexplored.

**Aim:**

Therefore, this study aimed to identify the causes of voluntarily reported medication incidents describing unintentional medication discrepancies occurring in care transitions. The secondary objective was to characterise the reported medication incidents regarding type of incident, medication involved, whether the incident reached the patient, and the transfer moment involved.

**Method:**

This cross-sectional study used data from the National Medication Incident Reporting database of the Dutch Institute for Rational Use of Medicine. This database contains medication incidents, mainly reported by healthcare professionals in hospitals. Incidents were included if they described an unintentional medication discrepancy in a care transition. The primary outcome was the cause of the reported incident independently determined by two researchers using the Prevention and Recovery Information System for Monitoring and Analysis model (PRISMA-Medical). Secondary outcomes were the incident type (e.g., omissions or dose discrepancies), the medication involved, whether the incident reached the patient, and the transfer moment. Descriptive statistics were used for data-analysis (frequencies and percentages).

**Results:**

A total of 32,261 incidents were reported in the study period, of which 992 (3.1%) met the inclusion criteria. Most incidents were attributed to human factors (n = 932; 94.0%), with verification errors (n = 492; 49.6%) and intervention errors (n = 246; 24.8%) being the most common. Organisational and technical causes accounted for 40 (4.0%) and 20 (2.0%) incidents, respectively. Omissions represented the most frequent type of discrepancies (n = 354; 36.2% of 978 reported discrepancy types), and cardiovascular medication was most frequently involved (n = 339; 24.9% of 1,360 reported medications). Among incidents with available data (n = 599), 444 (74.1%) reached the patient. Most incidents occurred at hospital admission (n = 679; 71.4% of 951 incidents with data available) and discharge (n = 229; 24.1%).

**Conclusion:**

Most reported incidents in care transitions were attributable to human error. These findings highlight the need for better preventive measures, e.g. patient-centred medication reconciliation to enhance patient safety.

**Supplementary Information:**

The online version contains supplementary material available at https://doi.org/10.1007/s11096-026-02189-x.

## Impact statements

- Reported medication incidents are a rich source to analyse the causes of unintentional medication discrepancies in care transitions.
- Most unintentional medication discrepancies, especially around a hospitalisation, are attributed to human factors, such as omitting a medication.
- Findings underline the importance of mitigating measures facilitating intuitive processes for the transfer of medication information.

## Introduction

Transitions of patients between healthcare settings are widely recognised as a patient safety risk [1, 2]. The transfer of medication information between healthcare professionals [3] can introduce medication discrepancies [4], potentially leading to adverse drug events [5, 6], hospital readmissions [7, 8] and increased healthcare costs [9]. The World Health Organization (WHO) report ‘Medication Safety In Transitions of Care’ emphasises the need to improve safety in care transitions, since almost all patients experience unintended medication discrepancies [1].

Since 2008, most Dutch hospitals have implemented medication reconciliation [10] to reduce unintentional medication discrepancies [11–14]. An unintentional medication discrepancy is an unintended difference between the medication used by the patient and the medication list of the healthcare provider [15]. In the Netherlands, medication reconciliation is often conducted by combining medication information from the Nationwide Medication Record System (NMRS) with a patient interview. The NMRS is a platform that exchanges dispensing data, after patient consent, from all community and outpatient pharmacies [16].

Despite efforts of medication reconciliation, studies continue to underscore the persistent problem of discrepancies [16]. Studies report incidences of patients with at least one unintentional medication discrepancy between 7 and 51% at hospital admission [17–19]. At hospital discharge, continuity of care issues were identified in 92% of discharge prescriptions, including administrative errors and counselling needs, with 31% attributable to discrepancies [10]. This was supported by another study reporting that 78% of patients experienced at least one discrepancy between their discharge prescription and the pharmacy records [20]. A substantially lower percentage of 5% was found in another study on discrepancies that remained undetected after medication reconciliation by hospital staff [18]. Causes of unintentional medication discrepancies included missing information (e.g. regarding dose changes or discontinued chronic medication) or errors during the execution of medication reconciliation (e.g. transcription errors) [10, 20].

To enhance patient safety and prevent medication-related harm, it is essential to understand the causes of unintentional medication discrepancies [21–23]. The WHO highlights that combining incident reporting with a thorough analysis of the causes is crucial to mitigate patient harm and improve learning from incidents [24]. Medication incident reporting systems are implemented in various countries [22], enabling healthcare professionals to voluntarily report incidents. However, analysis and learning from these incidents remain insufficient [21, 24]. Previous research on the causes of medication incidents was mostly restricted to medication errors in general, while medication incidents involving unintentional medication discrepancies during care transitions remain unexplored.

## Aim

Therefore, this study aimed to identify the causes of voluntarily reported medication incidents describing unintentional medication discrepancies occurring in care transitions. The secondary objective was to characterise the reported medication incidents regarding type of incident, medication involved, whether the incident reached the patient, and the transfer moment involved.

## Method

A cross-sectional study was conducted using data from the National Medication Incident Reporting (NMIR) database of the Dutch Institute for Rational Use of Medicine (IVM). Included were incidents reported between January 1, 2022, and August 1, 2023.

### Data source

The NMIR database is a reporting system in the Netherlands to which healthcare facilities and individual healthcare professionals can voluntarily report medication incidents. Most reported incidents (98%) originate from hospitals. The remaining incidents are reported by community pharmacists and general practitioners [25]. Incidents involving patient data were reported anonymously. If patient data were inadvertently included, an employee of the IVM anonymised the information. Consequently, we only received anonymised data. Healthcare professionals and institutions submitted reports via authenticated access. Healthcare professionals and institutions were pseudonymised before transfer. After reporting, the incidents are coded according to the phase of the medication process in which they occurred (e.g., prescribing, storage and logistics, administration/use, medication information transfer). If an incident fitted multiple categories, it was coded based on the initial phase of occurrence. This study analysed all incidents coded in the category medication information transfer.

### Eligibility criteria

For each incident, it was assessed whether an unintentional medication discrepancy occurred in a care transition. Care transitions were defined as transfers between settings, e.g., admission to or discharge from a hospital or another healthcare setting. Incidents were included if they involved an unintentional medication discrepancy caused by an error during medication reconciliation, during the transfer of medication information between healthcare professionals and/or in the written medication list provided to the patient. Incidents were excluded if they involved internal transfer within a single healthcare facility (e.g., intensive care unit (ICU) to ward), except for transfers from the emergency department to a ward (which reflects routine admission processes). Additionally, incidents were excluded if insufficient information was available to determine the cause.

### Data classification

The Prevention and Recovery Information System for Monitoring and Analysis (PRISMA)-Medical model was used to determine incident causes [26]. This model classifies causes on two levels, first as technical, organisational, human, or other factors, with human factors further subdivided into skill-, rule-, and knowledge-based behaviour. Second, these main categories are divided into twenty subcategories. The underlying contributing factor is determined by repeatedly asking ‘why’ [26]. The subcategory verification errors are similar and were defined as those occurring while performing medication reconciliation (e.g. reconciling a wrong dose or missing a stop date), whereas intervention errors were failures in the information or procedure on which the medication reconciliation depends (e.g. the NMRS was not consulted or outdated information was used). Although related, verification includes errors within the reconciliation process, whereas intervention includes errors in the process surrounding reconciliation. The initial 10% of the dataset was used to specify the subcategories by assigning incident examples (Online Resource 1). This process was carried out by two junior researchers supervised by a pharmacist of the IVM. Second, the complete dataset was analysed using the specified PRISMA-Medical model. The two junior researchers independently analysed all incidents. In case of disagreement, consensus was reached through discussion. If no consensus was reached, the incident was reviewed by a pharmacist of the IVM whose judgement was final. Throughout this process, the research group, which included the junior researchers, the IVM pharmacist, and two hospital pharmacists, engaged in continuous consultation on the inclusion and categorisation of incidents.

Interrater reliability between the junior researchers was calculated using percentage agreement and Cohen’s Kappa [27].

### Main outcome measures

The primary outcome was the cause of the reported incident. Secondary outcome measures were the type of incident, the involved medication classified according to the main anatomical or pharmacological groups of the Anatomical Therapeutic Chemical (ATC) classification [28], whether the incident reached the patient, and the specific transfer moment at which the incident occurred. Incident types were categorised as omissions, additions, discrepancies in dose or dose frequency, substitutions within the same or a different therapeutic group, discrepancies in duration of use, or in instructions for use. Transfers were categorised as admissions to or discharges from a hospital or healthcare facility, or transfers between healthcare facilities. Transfers were also classified by the type of healthcare facility (e.g., hospital, community pharmacy, or general practice). If this information was not reported, it was classified as unknown.

### Data analysis

No sample size calculation was performed because the study was designed as a descriptive analysis to provide insight into the causes of medication incident reports. A comprehensive time period of 1.5 years was selected to ensure the inclusion of a sufficient number of incidents.

The data were analysed using descriptive statistics in MS Office Excel version 16.82 (Microsoft, Redmond, WA, United States). The primary outcome was expressed as the percentage of reports with a certain cause among all unintentional medication discrepancy reports. The secondary outcomes were expressed as percentages of reports with available data, due to substantial missing data.

## Ethics approval

Ethical approval from the Medical Ethics Review Committee was not required due to the data’s retrospective, anonymous nature, in accordance with the Dutch Law. During analysis, the anonymised data were handled with strict confidentiality in private environments.

## Results

**Fig. 1:**
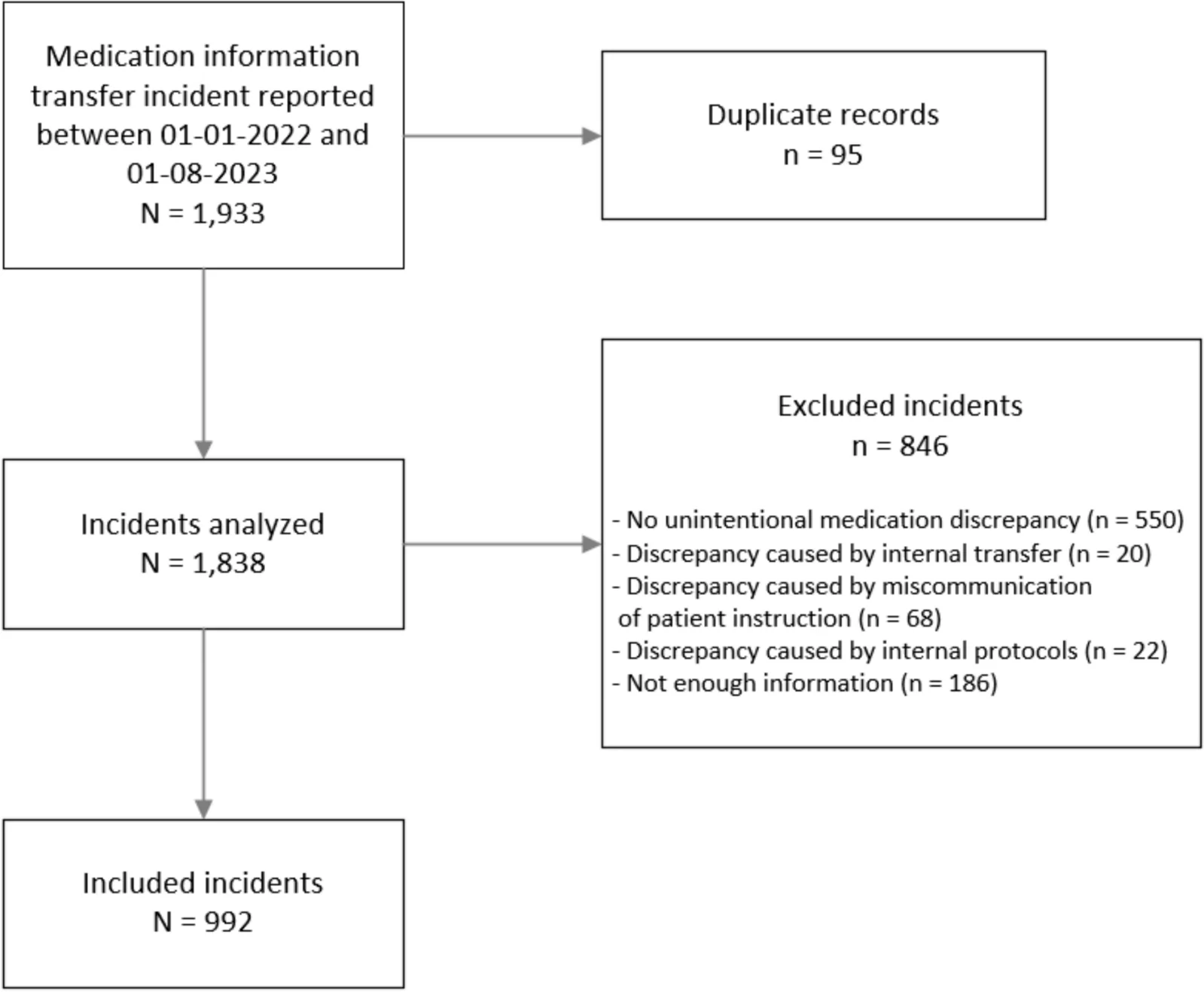
Flowchart inclusion of reported incidents

A total of 32,261 incidents were reported between January 1, 2022, and August 1, 2023. Of these, 1933 incidents were classified as medication information transfer incidents. Ninety-five were identified as duplicate reports, and 846 incidents were excluded based on the predefined exclusion criteria. The most common exclusion criteria were no unintentional medication discrepancies involved (n = 550; 65.0%), insufficient information to determine the cause (n = 186; 22.0%), and miscommunication between the healthcare professional and the patient (n = 68; 8.0%). In total, 992 of the incidents (3.1%) met the inclusion criteria and were included in the analysis (Fig. 1).

### Primary outcome measure

Interrater agreement and Cohen’s Kappa were calculated for the causes of 992 incidents assessed independently by two junior researchers. The interrater agreement was 73.5%, and the Cohen’s Kappa was 0.62 (CI 0.59–0.66; *p* < 0.001), indicating moderate agreement. Consensus could be reached in all 992 incidents.

The causes of the reported incidents are presented in Table 1. The majority of the incidents were attributed to human factors (n = 932; 94.0%). Within this category, verification errors were the most prevalent (n = 492; 49.6%). This category included errors by healthcare professionals during medication reconciliation (e.g., reconciling a wrong dose) and documentation errors, such as incorrect administration times caused by selecting the wrong box in the electronic health record (EHR). Intervention errors were the second most common category (n = 246; 24.8%). This category included errors resulting from deviations from the standard operating procedures for medication reconciliation, such as failing to consult the NMRS using outdated information, or not performing medication reconciliation altogether. The third most common category were coordination errors (n = 164; 16.5%). This category included incidents due to healthcare professionals working past each other, such as both physicians and pharmacy technicians conducting medication reconciliation, or errors arising if medication changes were not (clearly) communicated to the next healthcare professional.

**Table 1:** Causes of the incidents (n = 992)

Categories and subcategories	N (%)
*Technical*	
Design	14 (1.4%)
Construction	6 (0.6%)
*Organisational*	
Management Priorities	28 (2.8%)
Culture	7 (0.7%)
Protocols	3 (0.3%)
Transfer of knowledge	2 (0.2%)
*Human*	
Verification	492 (49.6%)
Intervention	246 (24.8%)
Coordination	164 (16.5%)
Qualifications	17 (1.7%)
Monitoring	11 (1.1%)
Slips	2 (0.2%)

The remaining incidents were attributed to organisational (n = 40; 4.0%) or technical causes (n = 20; 2.0%). Organisational errors arose when medication reconciliation was not performed due to a lack of staff for patient admissions outside regular office hours. Technical errors arose from EHR malfunctions, including error messages during medication list processing or interoperability failures between multiple EHR systems.

### Secondary outcome measures

Secondary outcomes are summarised in Table 2. Secondary outcome measures had substantial missing data. A single reported incident could contain multiple types of discrepancies or involved multiple medications. Therefore, the number of incidents in these categories (including reports with missing data) exceeds the total number of analysed incidents.

**Table 2:** Secondary outcome measures

Type of discrepancy^a^	N of incident types = 978^b^
Omission	354 (36.2%)
Dose and/or dose frequency discrepancy	266 (27.2%)
Addition	243 (24.8%)
Substitution of medication within the same or a different therapeutic group	62 (6.3%)
Duration of use discrepancy	27 (2.8%)
Instruction of use discrepancy	26 (2.7%)

Medication (ATC-classification)^a^	N of medications = 1360^b^
A (Alimentary tract and metabolism)	245 (18.0%)
B (Blood and blood forming organs)	257 (18.9%)
C (Cardiovascular system)	339 (24.9%)
D (Dermatologicals)	17 (1.3%)
G (Genito-urinary system and sex hormones)	24 (1.8%)
H (Systemic hormonal preparations, excluding sex hormones and insulins)	55 (4.0%)
J (Anti-infectives for systemic use)	46 (3.4%)
L (Antineoplastic and immunomodulating agents)	39 (2.9%)
M (Musculo-skeletal system)	36 (2.6%)
N (Nervous system)	219 (16.1%)
P (Antiparasitic products, insecticides and repellents)	2 (< 1.0%)
R (Respiratory system)	54 (4.0%)
S (Sensory organs)	24 (1.8%)
V (Various)	3 (< 1.0%)

Reached patient	N of incidents reached patient = 599^b^
Yes	444 (74.1%)
No	155 (25.9%)

Transfer moment	N of transfer moments = 951^b^
Hospital admission	679 (71.4%)
Previous healthcare setting unknown	648 (68.1%)
Transfer from other hospital	24 (2.5%)
Transfer from other healthcare setting﻿^c^	7 (< 1.0%)
Hospital discharge	229 (24.1%)
Subsequent healthcare setting unknown	174 (18.3%)
Transfer to community pharmacy	23 (2.4%)
Transfer to other healthcare setting^c^	32 (3.4%)
Admission and persistent after discharge	12 (1.3%)
Outpatient clinic	23 (2.4%)
Previous or subsequent healthcare setting unknown	19 (2.0%)
Transfer to other healthcare setting^c^	4 (< 1.0%)
Other transitions^c^	8 (< 1.0%)

Abbreviations: ATC-classification, Anatomical Therapeutic Chemical Classification

^a^ One incident can contain multiple types of discrepancies or multiple medications. Therefore, the number of incidents in these categories is higher than the total number of analysed incidents.

^b^Missing data: type of discrepancy (n = 105), medication ATC classification (n = 142), reached patient (n = 393), transfer moment (n = 41).

^c^Transitions from and to healthcare settings which are less than 1% of the incidents are combined into one category. An overview of these transitions is provided in Online Resource 2

#### Incident type

The incident type was missing in 105 reports (9.7%). Among reports with available data (n = 887), in total 978 incident types were reported. Omissions were most frequent (n = 354; 36.2%), followed by dose or dose frequency discrepancies (n = 266; 27.2%) and additions (n = 243; 24.8%).

#### Medication type

In 142 reports (9.5%), information regarding the medication involved was missing. For the 850 reports, in total 1360 medications were involved. The medication most commonly involved belonged to the following main ATC categories: cardiovascular system (n = 339; 24.9%), blood and blood forming organs (n = 257; 18.9%), and alimentary tract and metabolism (n = 245; 18.0%).

#### Reached patient

In 393 reports (39.6%), it was unclear whether the incident reached the patient. Among incidents with available data (n = 599), most reached the patient (n = 444; 74.1%), while 155 did not (25.9%).

#### Transfer moment

In 41 reports (4.1%), information regarding the transfer moment was unavailable. Among incidents with available data (n = 951), most were reported by hospital healthcare professionals, and occurred at the time of admission (n = 679; 71.4%) and discharge (n = 229; 24.1%). Additionally, a few incidents (n = 12; 1.3%) occurred at admission but were not detected during hospital stay or at discharge and therefore existed after discharge. A small number of incidents were reported during outpatient clinic visits (n = 23; 2.4%). Information regarding the previous or subsequent healthcare setting was unavailable in most cases. When this information was available (n = 98; 10.3%), hospital-to-hospital transfer (n = 24; 2.5%) and transfer from the hospital to community pharmacies (n = 23; 2.4%) were most common. A detailed overview of infrequent transfer moments is presented in Online Resource 2.

## Discussion

The results of this cross-sectional study show that human errors are frequently involved as a cause of reported unintentional medication discrepancies. Verification errors, intervention errors, and coordination errors were most prevalent, and the most common types of incidents were omissions, discrepancies in doses or dose frequencies, and additions.

Although previous literature has mainly focused on medication incidents in general [29–32], our results are in line with the study by Bosma et al*.* [33], investigating medication incidents during care transitions in the ICU*.* They reported that most incidents were due to communication errors (50%) and human performance (35%). Studies focusing on medication incidents in general found that most incidents were due to the patients’ poor understanding of medication use (35.6%), unawareness of medication changes (24.7%) [31], peak hours (34.8%), inexperienced personnel (32.4%) [32] or uncompleted or absent medication reconciliation (percentages not reported) [34]. While these categories overlap with the human errors in our study, the specific causes reported in these studies were less frequent in our dataset, likely because those studies focused on medication errors in general rather than medication discrepancies specifically. Other studies reporting medication errors found that most incidents were attributed to organisational factors (82–97%) [29, 30]. Bosma et al. [33] also reported substantially higher organisational errors rates than our findings. These differences can be explained by differences in classification methods [29, 33], the use of community pharmacy [30] or ICU data instead of hospital data [33], and the focus on all medication errors rather than discrepancies alone [29, 30].

In our study, the most frequent type of incident was omission (36%). Bosma et al*.* [33] reported higher percentages of omissions (52%), likely due to more stopped medications in the ICU. Alqenae et al*.* [29] found that most incidents were related to wrong or unclear doses (19%), omissions (13%), and wrong medication (10%), potentially reflecting differences in patient populations.

Moreover, we found that 74% of the incidents with available data, actually reached the patient. Similar results (81%) were reported by Bosma et al*.* [33]. Other studies report lower percentages of 12.6% [29] and 16.3% [30], potentially because they report actual patient harm, whereas we considered whether the incident reached the patient.

Cardiovascular medications were most frequently involved in our study. However, they were also among the most used medications in the Netherlands in 2022 [35], so this may reflect their high usage rather than an increased risk of discrepancies.

Most incidents (71.4%) were reported at admission. However, medication reconciliation is more routinely performed at admission, increasing opportunities to detect discrepancies [36]. Implementation of discharge reconciliation remains challenging due to communication difficulties and challenges embedding medication reconciliation in current clinical workflows [36, 37]. The higher percentage at admission may therefore partly reflect detection and reporting practices rather than true differences in risks.

### Strengths and limitations

This study has several strengths. To our knowledge, this is the first study to explore medication incidents specifically related to unintentional medication discrepancies. These findings provide actionable insights to prevent future incidents based on the identified causes. Additionally, the analysis is based on a large dataset, increasing reliability, and causes were independently classified by two researchers to minimise errors and bias.

Several limitations are also present. The incidents originate from a voluntary medication incident reporting system not intended for research, which may cause under- and selective reporting, since unrecognised or deemed unimportant incidents may be unreported. The extent of underreporting is unknown, as the actual number of incidents is unavailable. Incidents with technical or organisational causes are likely underestimated, such incidents often have recurring causes and are less likely to be reported repeatedly, and reports may lack sufficient information, limiting detailed analysis and which can result in an overrepresentation of human errors. Information in the reports was often limited, making it difficult to determine the actual cause and contributing to the moderate interrater reliability. To address this, a third author, experienced in analysing medication-incident reports, reviewed the incidents. Most disagreements occurred in the coordination, verification and intervention categories, and were resolved through discussion between the two researchers and the IVM pharmacist until consensus was reached. Insufficient information also caused substantial missing data, making it impossible to assess the causes. The same was true for the proportion of incidents reaching the patient, as this variable was missing for 39.6% of reports. The direction and magnitude of any resulting bias are unknown. Moreover, before receiving the data, the IVM had already classified incidents, which may have introduced misclassification and led to incorrectly in- or excluding incidents from our dataset, causing both under- and overestimation. Furthermore, most incidents originate from hospitals and a few from community pharmacies, so generalisability to all care transitions is unclear. Lastly, the severity of patient harm resulting could not be determined, due to insufficient information.

### Implications

Medication reconciliation is widely implemented in most hospitals in the Netherlands. However, discrepancies are a persistent challenge. Our findings demonstrate that the greatest potential for improvement lies in reducing human errors. This requires an approach across multiple domains.

Mitigation strategies should focus not only on training and enhancing human performance, but especially on improving technical and organisational processes that support these human factors. Such strategies should be integrated into both primary and secondary care settings.

Initially, the process of medication information transfer should be designed to facilitate human use. Both the process and supporting systems must be intuitive, ensuring clarity of actions without requiring extensive training, thereby minimising the risk of errors. Where feasible, manual interventions should be eliminated and replaced by automated solutions. A solution provided by previous literature is encouraging patients to take a more active role in medication information transfer. Active patient involvement can contribute to the detection or prevention of medication discrepancies, e.g. by reconciling their own medications [38] or by improved communication between patients and healthcare providers [39]. When feasible, patients should be granted greater autonomy in managing their medication information. However, this might not be feasible for all patients (e.g. patients in psychiatry or with reduced health literacy) [40].

Incident reporting in primary care should be particularly supported, given the limited number of incidents reported in this setting. Reporting of organisational and technical incidents should be supported, for example by incorporation predefined categories into incident reporting systems. This may help healthcare professionals better recognise these incident types. Further research is needed to develop and evaluate the effectiveness of preventative strategies.

## Conclusion

The findings of this study indicate that most reported unintentional medication discrepancies in care transitions are attributable to human errors. These findings highlight the need for new preventive measures, which should mainly focus on facilitating intuitive use of processes for transferring medication information and minimising human error risks. Encouraging incident reporting in primary care settings is also important to facilitate early identification and resolution of issues.

## Supplementary Information

Below is the link to the electronic supplementary material.

Supplementary file1 (DOCX 26 KB)

## Data Availability

The data that support the findings of this study are not openly available due to reasons of sensitivity and are available from the corresponding author upon reasonable request.
