# Supplementary material for: Causes and types of voluntarily reported unintentional medication discrepancies in care transitions: a cross-sectional study
Source: Int J Clin Pharm. 2026 Jul 18;48(5):2064–72. doi: 10.1007/s11096-026-02189-x (PMC13569517; doi:10.1007/s11096-026-02189-x)
Supplement: Supplementary file 1 [file 11096_2026_2189_MOESM1_ESM.docx]

**TITLE**

Causes and types of voluntarily reported unintentional medication discrepancies in care transitions – a cross-sectional study

**JOURNAL**

International Journal of Clinical Pharmacy

**AUTHORS**

Betsie Limmen ^a,b^, Joy van Broekhuizen ^c^, Rob Essink ^d^, Judith de Ruijter – van Dalem ^a,e^, Linda van Eikenhorst ^f^, Patricia M.L.A. van den Bemt ^g^, Fatma Karapinar – Çarkit ^a,b^

**AFFILIATIONS**

a) Department of Clinical Pharmacy and Toxicology, Maastricht University Medical Center+, Maastricht, The Netherlands
b) Department of Clinical Pharmacy Maastricht, CARIM Cardiovascular Research Institute Maastricht , Maastricht University, Maastricht, The Netherlands
c) Department of Clinical Pharmacy and Pharmacology, University Medical Centre Groningen, Groningen, The Netherlands
d) Dutch Institute for Rational Use of Medicine, Utrecht, The Netherlands
e) Department of Clinical Pharmacy, NUTRIM Institute of Nutrition and Translational Research in Metabolism, Maastricht University, Maastricht, The Netherlands
f) Department of Organization and Quality of Care, Netherlands Institute for Health Services Research (NIVEL), Utrecht, the Netherlands
g) Department of Clinical Pharmacy, University Medical Centre Utrecht, Utrecht, The Netherlands

**Corresponding author:**

Fatma Karapinar-Çarkit, PharmD, PhD

Address: Maastricht University Medical Centre+ (MUMC+), Maastricht, Department of Clinical Pharmacy and Toxicology, PO Box 5800, 6202AZ, Maastricht.

Tel: +31(0)43-3871881

E-mail: f.karapinar@mumc.nl

| Online Resource 1: Categories of causes according to Prevention and Recovery Information System for Monitoring and Analysis (PRISMA), specified for the study | | |
| --- | --- | --- |
| **Category** | **Definition original PRISMA model** | **Specification/Example** |
| **Technical** | | |
| External | Technical failures beyond the control and responsibility of the healthcare facility. | The electronic patient record has a functionality that is incorrectly built. |
| Design | Failures due to poor design of equipment, software, labels or forms. | Communication between the two systems fails. Example: a dosage isn't properly transferred from the Nationwide Medication Record System (NMRS) to the pharmacy information system. |
| Construction | Correct design, which is not constructed properly or is set up in inaccessible areas. | A discharge prescription that cannot be processed in the system due to an technical problem. |
| Material | Material defects not classified under design or construction. |  |
| **Organisational** | | |
| External | Failures at an organisational level beyond the control and responsibility of the healthcare facility, such as in another department or area (address by collaborative systems). | A health insurer stops reimbursing medication reconciliation. |
| Transfer of knowledge | Failures resulting from inadequate measures taken to ensure that situational or domain-specific knowledge or information is transferred to all new or inexperienced staff. | A new colleague is not (fully) trained in the medication reconciliation process. |
| Protocols | Failures relating to the quality and availability of the protocols within the department (too complicated, inaccurate, unrealistic, absent or poorly presented) | The medication reconciliation protocol is incorrect, missing, or not appropriate for practice. |
| Management Priorities | Internal management decisions in which safety is relegated to an inferior position when faced with conflicting demands or objectives. This is a conflict between production needs and safety. | No 24/7 medication reconciliation coverage. No medication reconciliation due to staffing issues or high workload. |
| Culture | Failures resulting from collective approach and its attendant modes of behaviour to risks in the healthcare facility. | Departments that do not take the patient and/or other departments into consideration.  Example: Medication orders are not prepared, and therefore, medication reconciliation cannot be completed, which is deemed unimportant by a healthcare professional. |
| **Human** | | |
| External | Human failures originating beyond the control and responsibility of the healthcare facility. This could apply to individuals in another department. |  |
| Knowledge-based behaviour | | |
| Knowledge-based behaviour | The inability of an individual to apply their existing knowledge to a novel situation. |  |
| Rule-based behaviour | | |
| Qualification | The incorrect fit between training or education of an individual and a particular task. | A healthcare provider/student is not authorized to perform medication reconciliation but did so anyway. Lack of (sufficient) knowledge of how the electronic health record works, resulting in medication not being entered (correctly). |
| Coordination | A lack of task coordination within a healthcare team in an organisation. | Healthcare providers work past each other.  Example: A physician performs medication reconciliation, but this is the pharmacy technician’s responsibility. Or medication is prescribed in the EHR before reconciliation is conducted.  Communication issues between healthcare providers.  Example: A detected discrepancy is communicated, but not followed up by the next healthcare provider.  An intentional change is not communicated, leaving the next healthcare provider unsure whether it is intentional. |
| Verification | The correct and complete assessment of a situation including related conditions of the patient and materials to be used before starting the intervention. | Errors occurring within the execution of the medication reconciliation, in other words, the healthcare provider performed the reconciliation but made a mistake in doing so.  An error because medication reconciliation is incompletely performed.  Example: an incorrect dosage, an omission, or a missing stop date.  Administrative errors during medication reconciliation.  Example: forgetting to check a box, leading to an incorrect adjustment of the medication administration time. |
| Intervention | Failures that result from faulty task planning and execution. | Errors in the information or procedure surrounding medication reconciliation; in other words, the healthcare provider did not follow the standard operating procedure or relied on inadequate information.  Medication reconciliation is not performed, resulting in a discrepancy.  Medication reconciliation is not performed according to protocol; for example, no medication list from the community pharmacy or the NMRS is not consulted.  Communication issue between the healthcare provider and the patient.  Example: the patient is not instructed about changes to their medication.  Errors caused by performing medication reconciliation using outdated medication data. Example: a healthcare provider performed medication reconciliation using an old medication list (and did not retrieve updated data from the NMRS). Or reconciliation is based on information copied from a previous, outdated reconciliation.  Errors caused by medication orders not being included in the administration record. Example: medication the patient used at home is continued by the pharmacy technician, but is neither reconciled nor approved by the physician. Or the medication from a previous hospital admission is not discontinued by the physician after reconciliation, leading to inappropriate continuation. |
| Monitoring | Monitoring a process or patient status. | Post-discharge medication management is not monitored or communicated to the next healthcare provider.  The appropriate method of medication dispensing is not properly verified.  Example: the pharmacy failed to notice that the medication should have been dispensed via medication roll packaging (Baxter roll).  Failure to recognize that the patient is unable to consult for medication reconciliation due to limited health literacy. |
| Skill-based behaviour | | |
| Slips | Failures in performance of highly developed skills. | A typo result in the selection of the wrong medication |
| Tripping | Failures in whole body movements. These errors are often referred to as “slipping, tripping, or falling”. |  |
| **Other Factors** | | |
| Patient-related | Failures related to patient characteristics or conditions, which are beyond the control of staff and influence treatment. |  |
| Other | Failures that cannot be classified in any other category. |  |
| NMRS: Nationwide Medication Record System; PRISMA: Prevention and Recovery Information System for Monitoring and Analysis | | |

| Online Resource 2: Transfer moments | |
| --- | --- |
| **Transfer moment** | **Number of reports** |
| Hospital admission from: |  |
| Primary care^a, b, c, d^ | 6 (0.6%) |
| Hospital discharge to: |  |
| Primary care^c, d, e, f, g, h^  Secondary care^i, j^ | 20 (2.0%)  12 (1.2%) |
| Outpatient clinic to: |  |
| Primary care^a, d^  Secondary care^i^ | 1 (0.1%)  3 (0.3%) |
| Other transitions: |  |
| Primary care^a, e^ to primary care^a, d^  Secondary care^i^ to primary care^c, d^ | 4 (0.4%)  4 (0.4%) |
| Values are presented as frequencies and percentages.  ^a^ Community pharmacy ^b^ Disability care ^c^ Nursing home ^d^ Home nursing ^e^ General practitioner ^f^ Hospice ^g^ Rehabilitation care ^h^ Addiction care ^i^ Anticoagulation clinic ^j^ Other hospital | |
